# Supplementary material for: A Toxoplasma gondii O-glycosyltransferase that modulates bradyzoite cyst wall rigidity is distinct from host homologues
Source: Nat Commun. 2024 May 6;15:3792. doi: 10.1038/s41467-024-48253-w (PMC11074326; doi:10.1038/s41467-024-48253-w)
Supplement: Supplementary file 1 — Supplementary Information [file 41467_2024_48253_MOESM1_ESM.pdf]

Supplementary Information

A *Toxoplasma gondii* O-glycosyltransferase that modulates bradyzoite cyst wall rigidity is distinct from host homologues

Pranav Kumar[1], Tadakimi Tomita[2], Thomas Gerken[3], Collin Ballard[3], Yong Sok Lee[4], Louis M. Weiss[2,5], Nadine L. Samara[1]

Supplemental results

Data processing, scaling and space group determination

The x-ray diffraction data were processed and scaled in orthorhombic space groups in HKL2000. The structures were solved using the Phaser software using a search method that performs molecular replacement using all possible space groups within a point group. The software assigned the P22121 space group for 6 of the 8 structures: Apo, Mn^{2+}-soaked, pH-soaked, CST1.4, Muc5AC-3,13 , Muc5AC-13. Similar results were obtained using MolRep (CCP4). These structures contain 1 molecule/asymmetric unit (Table S1 and S4). Changing the setting from P22121 to P212121 results in an ambiguous map. Phaser selected the P212121 space group for the remaining 2 structures: TxgGalNAc-T3 bound to SRS13.2 and TxgGalNAc-T3 bound to Muc5AC-3, which contain 2 complexes in the asymmetric unit (Table S1). The commonality is that they both contain a peptide with GalNAc at the N-terminus (Muc5Ac-3 and SRS13.2, Table S1). Given the lack of electron density for the peptides in these structures, it is not clear why the asymmetric unit symmetry is not “broken” in these structures.

Supplemental figures and tables

a

	TxgGalNAc-T2	TxgGalNAc-T1	TxgGalNAc-T5	TxgGalNAc-T4	**TxgGalNAc-T3**
TxgGalNAc-T2	100	27	29	30	**24**
TxgGalNAc-T1	27	100	34	37	**34**
TxgGalNAc-T5	29	34	100	43	**40**
TxgGalNAc-T4	30	37	43	100	**48**
TxgGalNAc-T3	24	34	40	48	**100**

Sequence Identity (%)

25 100

b

	GalNAc-T8	GalNAc-T18	GalNAc-T9	GalNAc-T19	TxgGalNAc-T3	GalNAc-T10	GalNAc-T17	GalNAc-T5	GalNAc-T7	GalNAc-T15
GalNAc-T8	100	48	45	44	31	29	28	31	29	30
GalNAc-T18	48	100	51	51	29	30	31	32	28	31
GalNAc-T9	45	51	100	62	32	31	31	34	32	32
GalNAc-T19	44	51	61	100	31	34	32	34	31	33
**TxgGalNAc-T3**	31	29	32	31	100	31	29	32	30	31
GalNAc-T10	29	30	31	34	31	100	72	39	38	36
GalNAc-T17	28	31	31	32	29	72	100	38	38	34
GalNAc-T5	31	32	34	34	32	39	38	100	32	34
GalNAc-T7	29	28	32	31	30	38	38	32	100	30
GalNAc-T15	30	31	32	33	31	36	34	34	30	100
GalNAc-T11	33	32	35	34	34	37	37	36	35	35
GalNAc-T20	34	33	33	33	34	36	38	39	35	38
GalNAc-T3	35	34	35	34	33	36	36	38	32	35
GalNAc-T6	35	35	37	35	33	36	36	37	35	36
GalNAc-T4	34	33	36	36	35	39	39	39	35	39
GalNAc-T12	30	34	34	34	34	39	38	37	32	42
GalNAc-T1	34	34	35	36	33	42	41	38	38	36
GalNAc-T13	35	34	37	36	34	42	40	37	37	35
GalNAc-T2	32	34	34	35	35	37	36	38	31	33
GalNAc-T14	32	31	33	34	33	39	38	39	34	33
GalNAc-T16	34	33	36	34	33	39	38	39	32	32

	GalNAc-T11	GalNAc-T20	GalNAc-T3	GalNAc-T6	GalNAc-T4	GalNAc-T12	GalNAc-T1	GalNAc-T13	GalNAc-T2	GalNAc-T14	GalNAc-T16
GalNAc-T8	33	34	35	35	34	30	34	35	32	32	34
GalNAc-T18	32	33	34	35	33	34	34	34	34	31	33
GalNAc-T9	35	33	35	37	36	34	35	37	34	33	36
GalNAc-T19	34	33	34	35	36	34	36	36	35	34	34
**TxgGalNAc-T3**	34	34	33	33	35	34	33	34	35	33	33
GalNAc-T10	37	36	36	36	39	39	42	42	37	39	39
GalNAc-T17	37	38	36	36	39	38	41	40	36	38	38
GalNAc-T5	36	39	38	37	39	37	38	37	38	39	39
GalNAc-T7	35	35	32	35	35	32	38	37	31	34	32
GalNAc-T15	35	38	35	36	39	42	36	35	33	33	32
GalNAc-T11	100	54	38	38	38	37	43	43	38	40	40
GalNAc-T20	54	100	36	36	39	38	41	41	38	39	38
GalNAc-T3	38	36	100	65	46	44	44	44	39	41	41
GalNAc-T6	38	36	65	100	46	45	44	46	41	41	40
GalNAc-T4	38	39	46	46	100	57	43	44	40	40	41
GalNAc-T12	37	38	44	45	57	100	44	44	41	37	38
GalNAc-T1	43	41	44	44	43	44	100	84	42	39	38
GalNAc-T13	43	41	44	46	44	44	84	100	42	41	39
GalNAc-T2	38	38	39	41	40	41	42	42	100	49	52
GalNAc-T14	40	39	41	41	40	37	39	41	49	100	55
GalNAc-T16	40	38	41	40	41	38	38	39	52	55	100

Sequence Identity (%)

28 100

Supplementary Fig. 1 | Sequence identity heat maps. a Comparison of *T. gondii* GalNAc-T isoenzymes. **b** Comparison of TxgGalNAc-T3 to human homologues, GalNAc-T1 to T20. Results were generated in Clustal Omega.

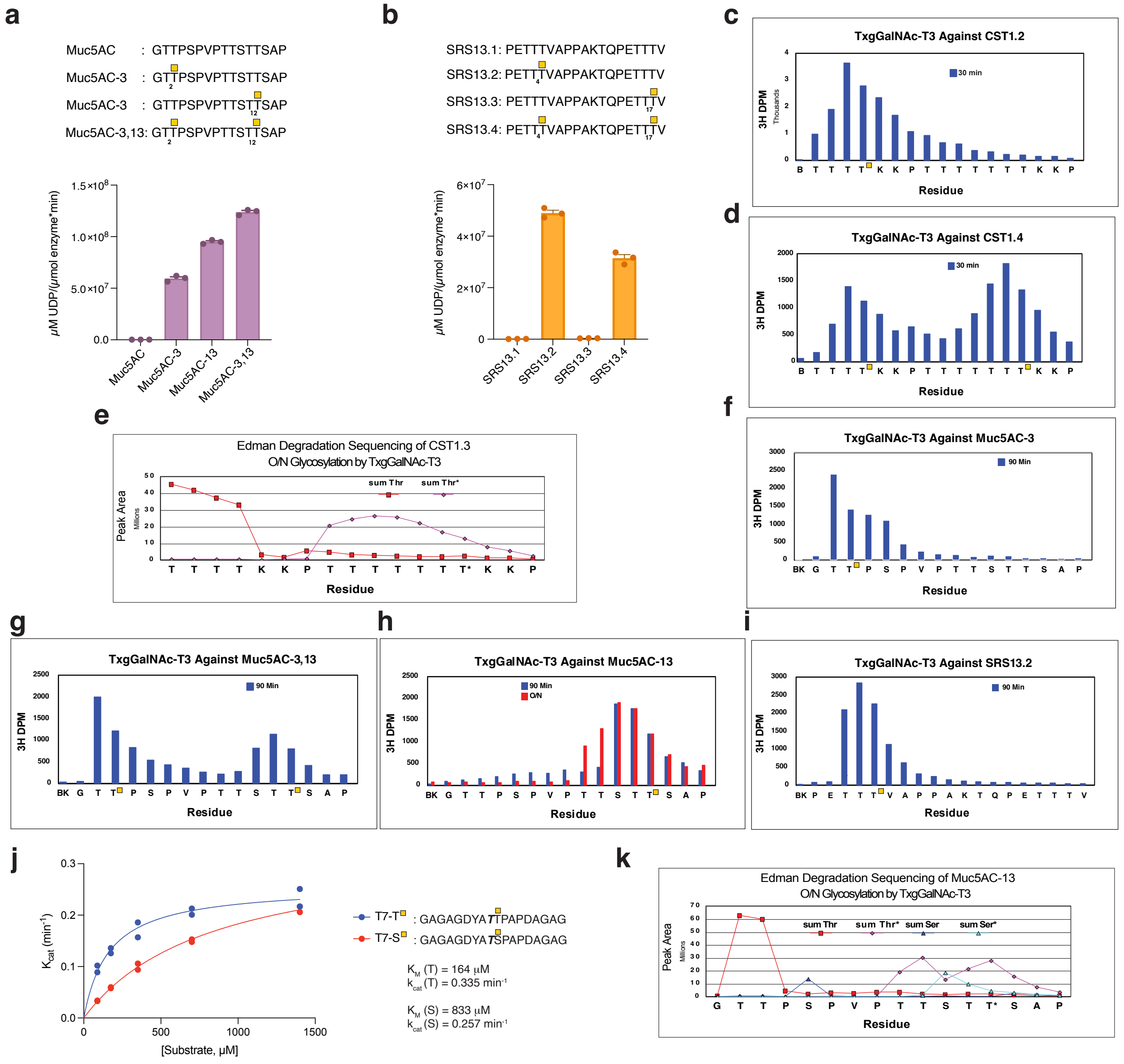

Supplementary Fig. 2 | Substrate preferences of TxgGalNAc-T3. a TxgGalNAc-T3 O-glycosylation of Muc5AC (glyco)peptides. **b** TxgGalNAc-T3 O-glycosylation of SRS13 (glyco)peptides, where n=3 biologically independent experiments performed in triplicate each time (9 technical replicates). The error bars in **a** and **b** represent the standard error

of the mean (SEM). **c and d** ^{3}H-GalNAc content of Edman sequencing confirming sequential glycosylation of CST1 glycopeptides after 30 min reactions (Note the presence of Edman sequencer lag of the ^{3}H-GalNAc-O-Thr-PTH derivatives in **c-d**). **e** Analysis of the Edman sequencing chromatogram of CST1.3 incubated overnight with TxgGalNAc-T3 demonstrating the nearly full glycosylation of acceptor residues N-terminal of the initial glycosylated Thr. Sum Thr represents the sum of Thr-PTH, dehydro-Thr-PTH & dehydro-Thr-DTT-PTH derivatives, sum Ser represents the sum of Ser-PTH and dehydro-Ser-DTT-PTH derivatives. Sum T* and Sum S* represents the sum of two Ser-O-GalNAc and Thr-O-GalNAc PTH diastereomers, respectively, that migrate differently on the column. **f - h** ^{3}H-GalNAc content of Edman sequencing confirming sequential glycosylation of Muc5AC glycopeptides after a 90 min reaction. **g** Like CST1.3, Muc5AC-13 becomes densely O-glycosylated after an overnight reaction. **i** Edman peptide sequencing confirm O-glycosylation of the SRS13.2 mono-glycopeptide. **j** Kinetic assay comparing Thr-O-GalNAc and Ser-O-GalNAc glycopeptides showing lower K_M for the Thr-O-GalNAc glycopeptide, suggesting tighter binding and stronger preference for Thr-O-GalNAc over Ser-O-GalNAc for TxgGalNAc-T3, n=2 biological replicates. **k** Analysis of the Edman sequencing chromatogram of CST1.3 incubated overnight with TxgGalNAc-T3 demonstrating the nearly full glycosylation of acceptor residues N-terminal of the initial glycosylated Thr. Sum Thr represents the sum of Thr-PTH, dehydro-Thr-PTH & dehydro-Thr-DTT-PTH derivatives, sum Ser represents the sum of Ser-PTH and dehydro-Ser-DTT-PTH derivatives. Sum T* and Sum S* represents the sum of two Ser-O-GalNAc and Thr-O-GalNAc PTH diastereomers, respectively, that migrate differently on the column. Source data are provided as a Source Data file.

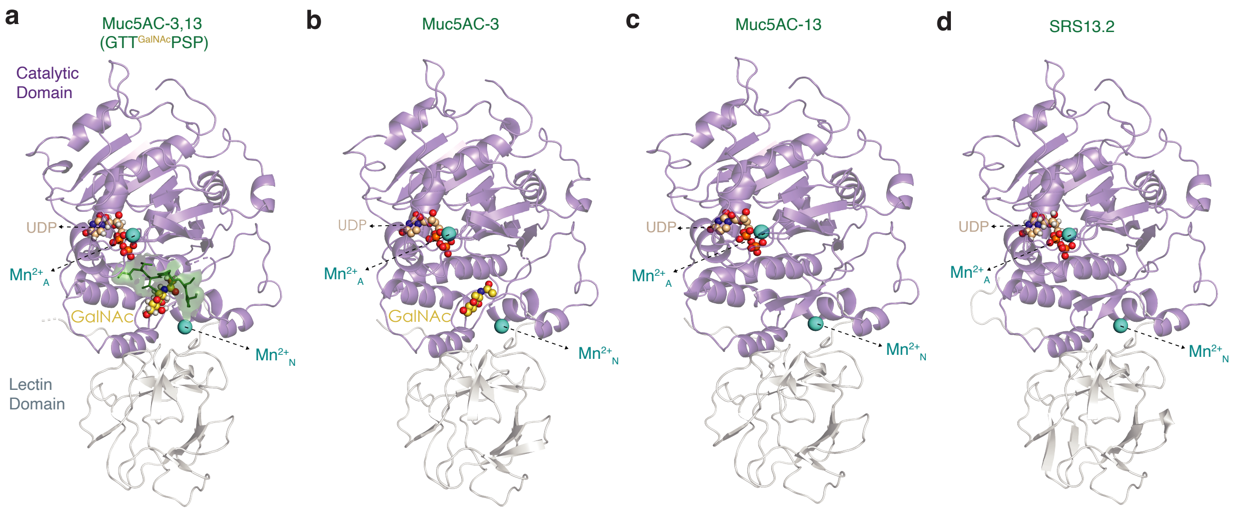

Supplementary Fig. 3 I X-ray crystal structures of TxgGalNAc-T3 in complex with various glycopeptides. The catalytic domain is lavender, UDP is wheat, Mn^{2+} is aquamarine, the C-terminal lectin domain is light grey, the peptides are green, and GalNAc is yellow. **a** The Muc5AC-3,13 bound TxgGalNAc-T3 with strong density for the N-terminal portion of the glycopeptide. Peptide density is weak in **b-d**, but GalNAc density is observed in the complex with Muc5AC-3 (**b**) suggesting the peptide is present but weakly bound in the crystal.

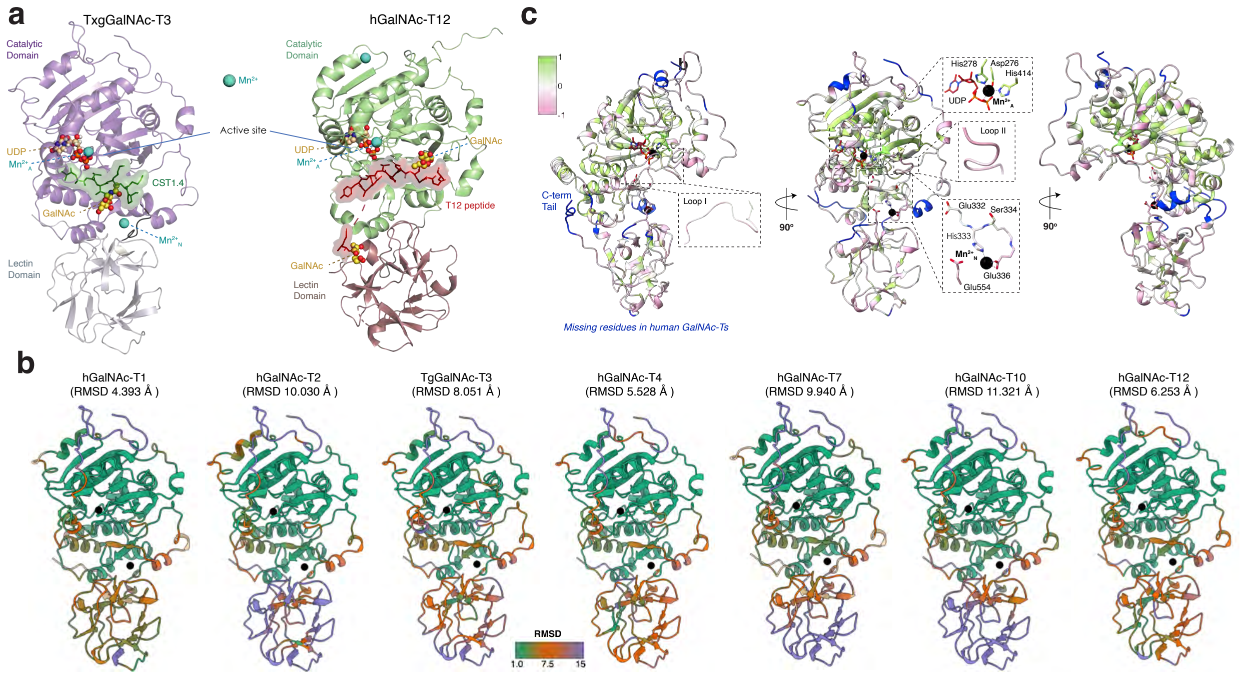

Supplementary Fig. 4 | TxgGalNAc-T3 structure analysis. a Comparison of TxgGalNAc-T3: CST1.4 co-crystal complex (catalytic domain is lavender, lectin domain is grey, peptide is green, and Mn^{2+} is aquamarine) to the human GalNAc-T12: T12 peptide co-crystal complex (catalytic domain is green, lectin domain is copper, peptide is red, and Mn^{2+} is aquamarine). The overall domain architecture and active sites are conserved, with a N-terminal catalytic domain tethered to a C-terminal lectin domain by a short linker. The acceptor Thr is similarly positioned in the active site for GalNAc transfer from UDP-GalNAc. TxgGalNAc-T3 has a second metal site (Mn^{2+}_N, shown in aquamarine). **b** RMSD calculations comparing the structure of TxgGalNAc-T3 to metazoan structures, including human (h) GalNAc-T1 (PDB ID 1XHB), -T2 (PDB ID 5AJP), -T4 (PDB ID 6H0B), -T7 (PDB ID 6IWR), -T10 (PDB ID 2D7R), and -T12 (PDB ID 6PXU), and *Taeniopygia guttata* (Tg) GalNAc-T3 (PDB ID 6S24) superposed over the structure of TxgGalNAc-T3. Calculations, analyses, and figures were generated with ChimeraX. **c** Sequence similarity calculation comparing TxgGalNAc-T3 to human isoenzymes (GalNAc-T1 to GalNAc-T20) showing conserved features such as the active site residues in green, non-conserved features

such as the second metal site and an active site loop II in pink, and residues absent in human isoenzymes in blue, such as the TxgGalNAc-T3 C-terminal tail. Calculations, analyses, and figures were generated with ChimeraX.

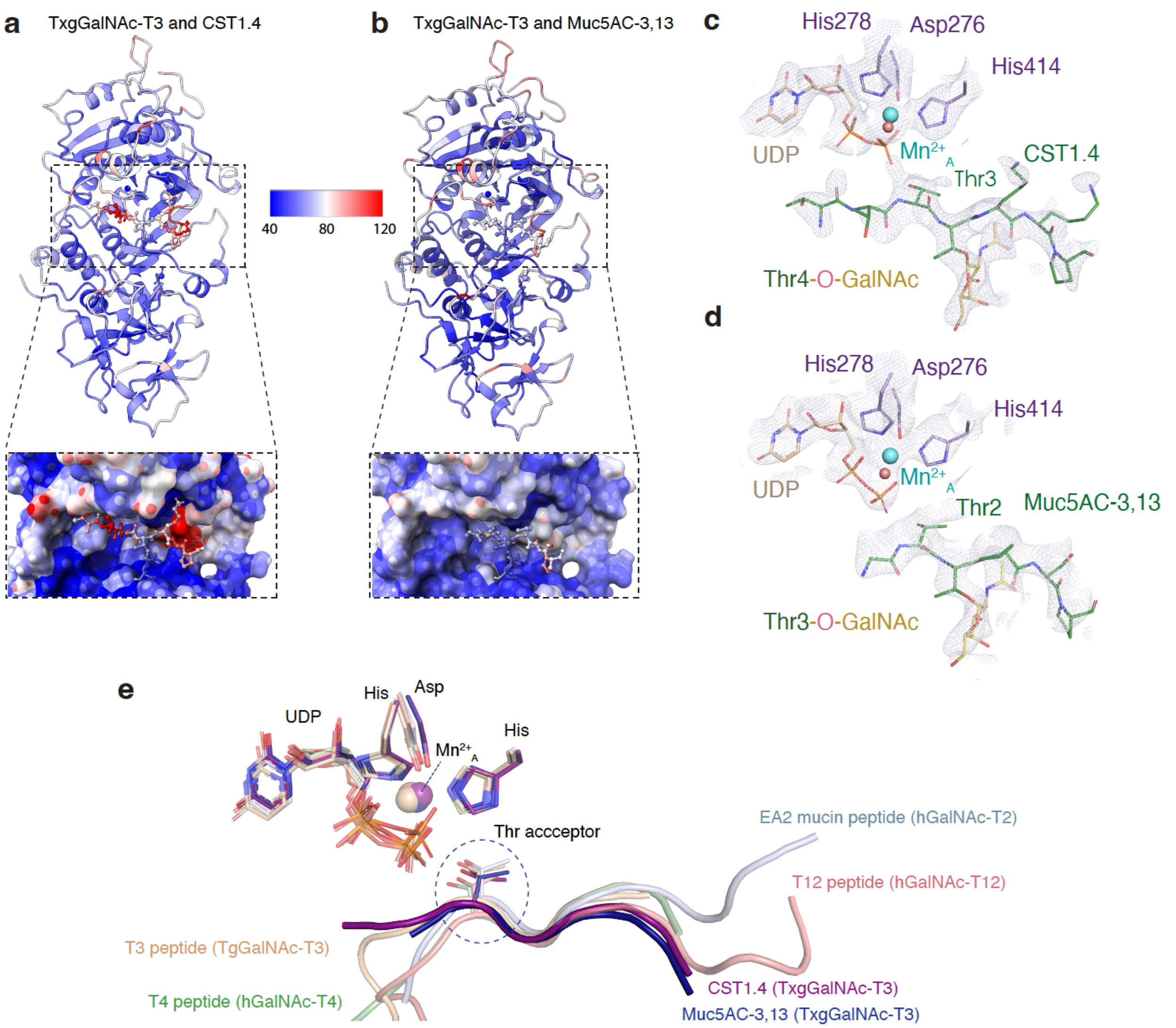

Supplementary Fig. 5 I TxgGalNAc-T3 peptide binding analysis. a B-factor visualization for TxgGalNAc-T3 bound to CST1.4 and **b** B-factor visualization for TxgGalNAc-T3 bound to Muc5AC-3,13. The scale shows b-factor of 40 (blue) to 120 (red). Calculations were performed during PHENIX refinement, while analyses and figures were generated with ChimeraX. **c** Unbiased map (grey) calculated for TxgGalNAc-T3 bound to CST1.4 contoured at 0.8σ. To calculate the map, we deleted the peptide and active site residues and performed molecular replacement with Phaser (PHENIX). **d** Similar to **c** but for the structure containing TxgGalNAc-T3 bound to Muc5AC-3,13. **e** A superposition of CST1.4 (purple) and Muc5AC-3,13 (dark blue) from TxgGalNAc-T3 complexes with peptides bound to metazoan isoenzymes from structures: hGalNAc-T2

(light blue, PDB ID 2FFU), TgGalNAc-T3 (beige, PDB ID 6S24), hGalNAc-T4 (green, PDB ID 6H0B), and hGalNAc-T12 (salmon, PDB ID 6PXU).

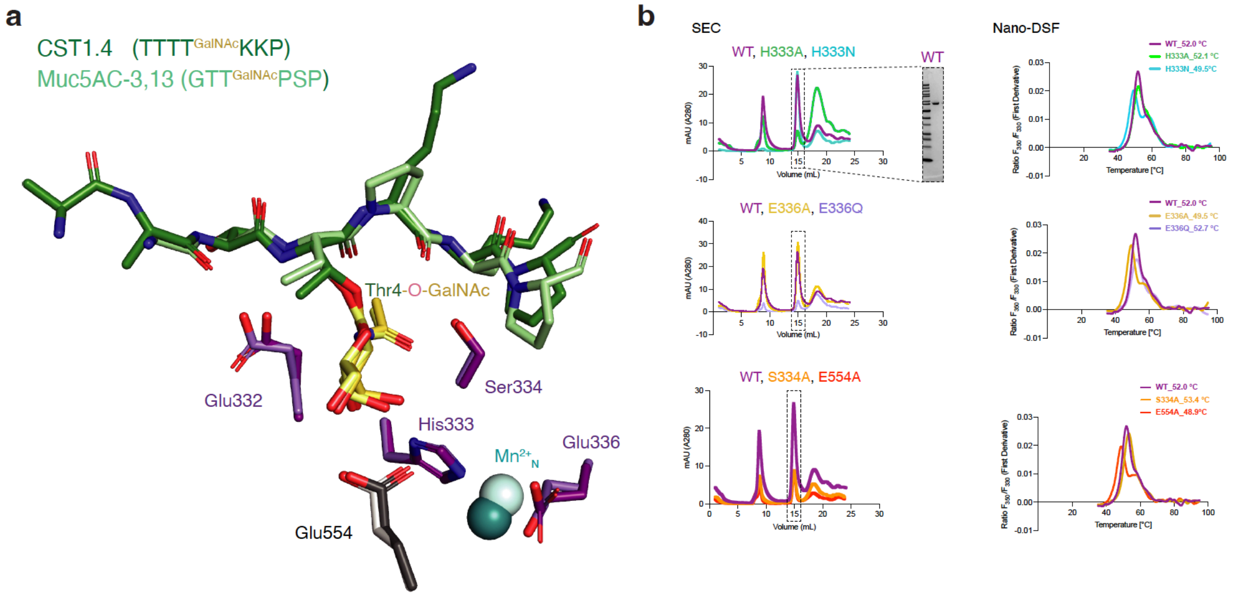

Supplementary Fig. 6 | Analysis of the second metal site variants. a CST1.4 (dark green) and Muc5AC-3,13 (light green) have distinct peptide sequences, but similarly bind to TxgGalNAc-T3 (sidechains shown in purple) with GalNAc positioned in the pocket coupled to the second Mn^{2+} site (N). For both peptides, GalNAc dictates substrate binding and recognition. **b** Size exclusion chromatography (SEC) and Nano-DSF data showing first derivatives (F350/F330) vs. Temperature (°C) for the TxgGalNAc-T3 second metal site variants. Source data are provided as a Source Data file.

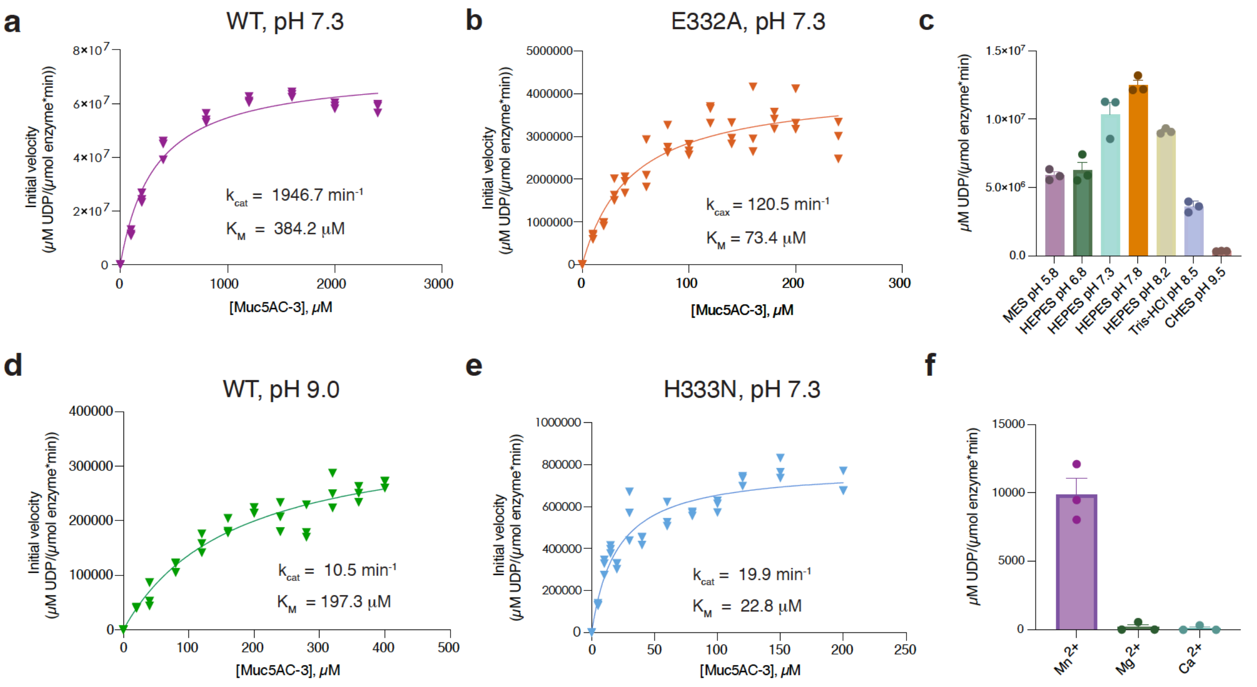

Supplementary Fig. 7 | Assessing the activity of TxgGalNAc-T3. **a** TxgGalNAc-T3^{WT} activity assay at pH 7.3. **b** TxgGalNAc-T3^{E332A} activity assay at pH 7.3 shows a ~16-fold reduction in k_{cat} and ~5-fold decrease in K_M compared to TxgGalNAc-T3^{WT}. **c** pH dependence of TxgGalNAc-T3 activity shows optimal activity at around pH 7.3-8.2. **d** TxgGalNAc-T3^{WT} activity assay at pH 9.0 shows a ~185-fold reduction in k_{cat} with and ~2-fold reduction in K_M compared with TxgGalNAc-T3^{WT} activity at pH 7.3. **e** TxgGalNAc-T3^{H333N} activity assay at pH 7.3 shows a ~100-fold reduction in k_{cat} and ~17-fold decrease in K_M compared to TxgGalNAc-T3^{WT}. **f** Comparison of TxgGalNAc-T3^{WT} activity in the presence of Mn^{2+}, Mg^{2+}, and Ca^{2+}. For all assays, n=3 biologically independent experiments performed in triplicate each time (9 technical replicates). The error bars in **c** and **f** represent the standard error of the mean (SEM). Source data are provided as a Source Data file.

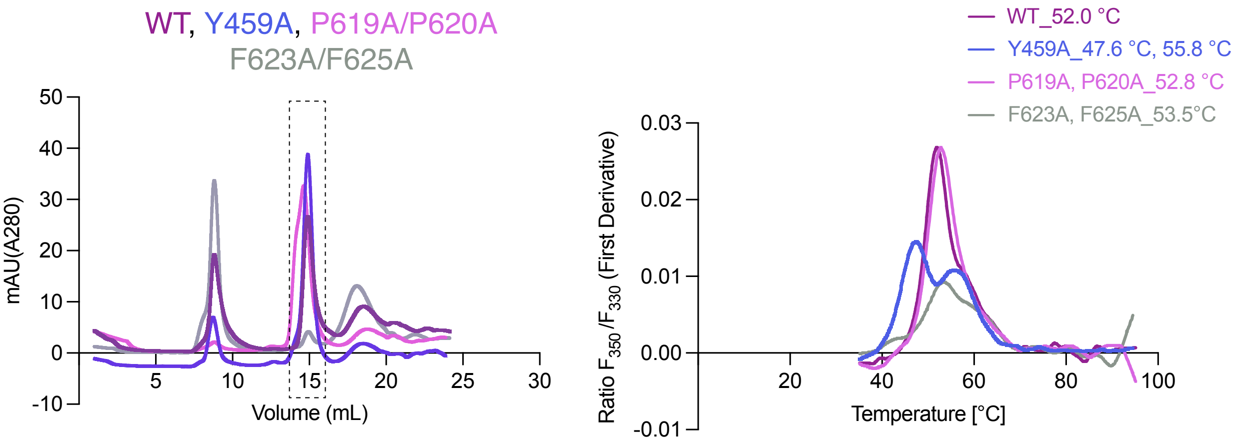

Supplementary Fig. 8 | Analysis of C-terminal tail variants. Size exclusion chromatography (SEC) and Nano-DSF data showing first derivatives (F350/F330) vs. Temperature (°C) for the TxgGalNAc-T3 C-terminal tail variants. Source data are provided as a Source Data file.

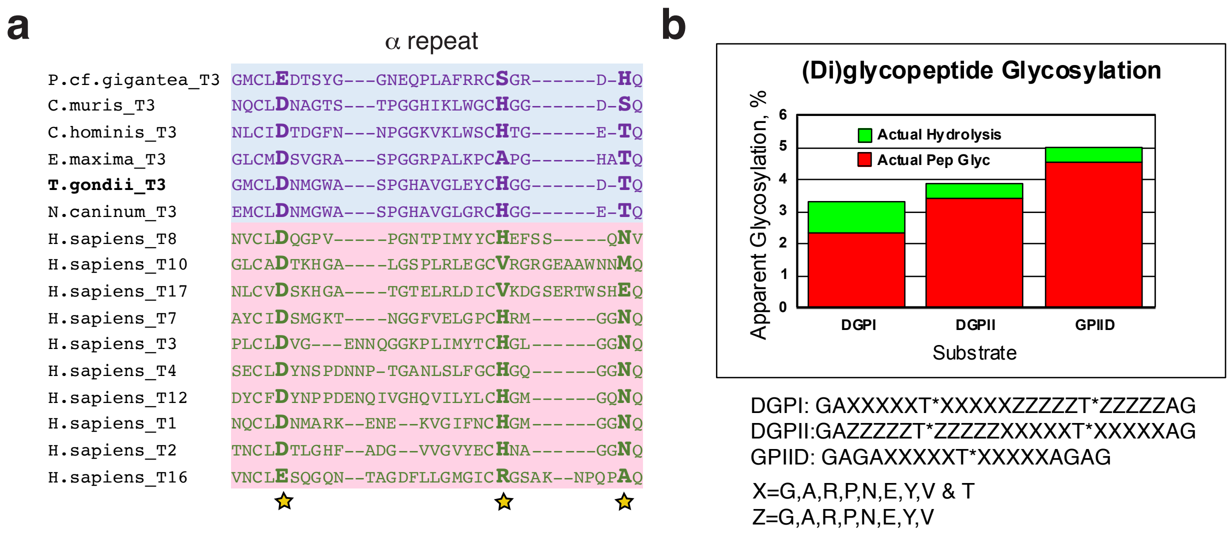

Supplementary Fig. 9 | Function of the TxgGalNAc-T3 lectin domain. a Sequence alignment comparing the α repeat of the lectin domains of TxgGalNAc-T3 and apicomplexan homologues to the lectin domain of human GalNAc-Ts. TxgGalNAc-T3 contains Asp and His required for GalNAc binding, but not Asn (yellow starred residues). Instead, this position is occupied by Thr. **b** Probing lectin domain mediated long-range effects on activity using di-glycopeptide libraries containing GalNAc positioned N-terminal (DGPI) or C-terminal (DGPII) to the acceptor. There is no significant enhancement compared to the GPIID mono-glycopeptides, providing little evidence of lectin domain involvement in activity. Source data are provided as a Source Data file.

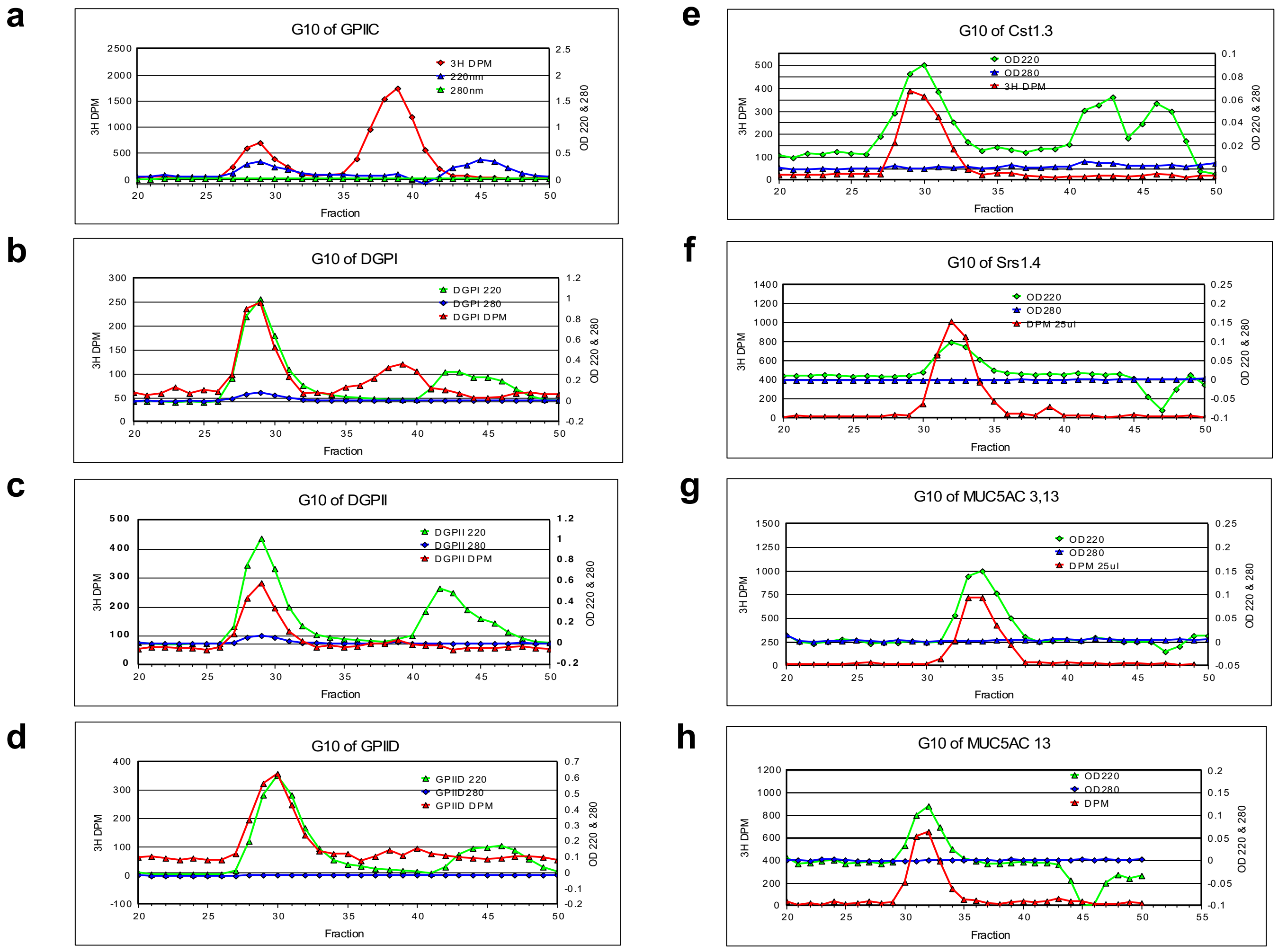

Supplementary Fig. 10 l Sephadex G10 column chromatography of the post Dowex 1X8 reactions of TxgGalNAc-T3 against different representative acceptor glycopeptides, demonstrating various extents of UDP-[^{3}H]GalNAc hydrolysis. ^{3}H-glycopeptide products appear between fractions 27-37 while free ^{3}H-GalNAc (hydrolysis) appear between fractions 35-43 depending on the column used. Overnight reaction for **a** GPIIC and 5-hour reactions for **b** DGPI, **c** DGPII and **d** GPIID. Thirty-minute reaction for **e** CST1.3, and 90-minute reactions for **f** SRS1.4 and **g** Muc5AC-3. Overnight reaction for **h** Muc5AC-3,13. ^{3}H DMP (from 25-50 ml aliquots of each ~1 ml fraction) are shown as the red plots, while OD220 and OD280 are shown as the green and blue plots, respectively. Source data are provided as a Source Data file.

Table S1: Data collection and refinement statistics: glycopeptide bound substrates

	TxgGalNAc-T3: SRS13.2, Mn^{2+}, UDP-GalNAc-F, pH 9.5	TxgGalNAc-T3: CST1.4, Mn^{2+}, UDP-GalNAc-F, pH 9.5	TxgGalNAc-T3: Muc5AC-3, Mn^{2+}, UDP-GalNAc-F, pH 9.5	TxgGalNAc-T3: Muc5AC-13, Mn^{2+},UDP-GalNAc-F, pH 9.5	TxgGalNAc-T3: Muc5AC-3,13, Mn^{2+},UDP-GalNAc-F, pH 9.5
	PDB ID **8UJG**	PDB ID **8UJH**	PDB ID **8UJF**	PDB ID **8UJE**	PDB ID **8UI6**
**Data collection**					
Energy (λ)	1 Å	1 Å	1 Å	1 Å	1 Å
Space group	P 21 21 21	P 2 21 21	P 21 21 21	P 2 21 21	P 2 21 21
Molecules/asymmetric unit	2	1	2	1	1
Cell dimensions					
a, b, c (Å)	66.614, 122.624, 165.655	61.722, 66.833, 166.047	66.233, 123.693, 165.724	62.409, 65.986, 166.671	61.252, 66.913, 163.581
α, β, γ (°)	90, 90, 90	90, 90, 90	90, 90, 90	90, 90, 90	90, 90, 90
Resolution (Å) [1]	29.5 - 2.27 (2.36 - 2.27)	29.4 - 2.65 (2.74 - 2.65)	20.0 - 2.87 (2.98 - 2.87)	19.9 - 2.50 (2.59 - 2.50)	20.0 - 2.65 (2.75 - 2.65)
R_{pim} [1]	0.081 (0.682)	0.120 (0.822)	0.136 (0.697)	0.072 (0.598)	0.060 (0.370)
$I/\sigma I$ [1]	12.0 (1.44)	7.6 (0.93)	5.01 (0.87)	10.3 (1.2)	14.8 (2.3)
$CC^{1/2}$	0.991 (0.504)	0.949 (0.306)	0.95 (0.377)	1.00 (0.43)	0.989 (0.732)
Completeness (%) [1]	99.5 (99.3)	99.6 (96.0)	97.2 (90.5)	99.8 (96.1)	100 (99.8)
Redundancy [1]	7.0 (6.8)	6.5 (4.3)	6 (5.4)	4.9 (3.1)	6.9 (4.7)
No. unique					
reflections [1]	62158 (3034)	20609 (974)	30811 (1402)	24551 (1164)	20128 (961)
**Refinement**					
R_{work} / R_{free}	19.2/23.7	19.1/ 23.5	21.7/ 27.4	18.2/ 23.0	17.4/ 23.2
No. atoms					
Protein	17081	8292	16463	8491	8328
Mn^{2+}	4	2	4	2	2
UDP	72	36	72	36	36
Peptide / 2-acetamido-2-deoxy-β-D-galactopyranose	42/28	100/28	53/28	0	72/28
Water / Solvent	266/99	20/12	25/0	30/19	20/31
Average B-factors	48.39	51.16	51.88	59.08	58.74
Macromolecules	49.93	51.08	55.70	58.55	58.74
Solvent[2]	44.93	44.24	37.93	52.31	47.83
Ligands	74.83	60.93	54.14	84.90	61.78
R.m.s deviations					
Bond lengths (Å)	0.003	0.003	0.0013	0.005	0.002
Bond angles (°)	0.67	0.55	1.48	0.65	0.50

[1] Data in the highest resolution shell is shown in the parenthesis.

[2] Water, Di(HydroxyEthyl)Ether and glycerol

Table S2: TxgGalNAc-T3 protein constructs and yields

Enzyme	Approximate Yield (mg/L)
TxgGalNAc-T3 WT	1.1
TxgGalNAc-T3_H333A	0.02
TxgGalNAc-T3_H333N	0.8
TxgGalNAc-T3_S334A	0.3
TxgGalNAc-T3_E336A	0.25
TxgGalNAc-T3_E336Q	0.42
TxgGalNAc-T3_E554A	0.8
TxgGalNAc-T3_ I320P	0.3
TxgGalNAc-T3_ P619, P620A	0.22
TxgGalNAc-T3_ F623, F625A	0.26
TxgGalNAc-T3_ Y459A	0.3
TxgGalNAc-T3_ E332A	0.25
TxgGalNAc-T3_ E332W	0.3
TxgGalNAc-T3_ E332F	0.2
TxgGalNAc-T3_ E332N	0.2
TxgGalNAc-T3_ E332Q	0.2
TxgGalNAc-T3_ E332D	0.15

Table S3: TxgGalNAc-T3 kinetic data

		WT(pH 7.3)	WT (pH 9.0)	H333N (pH 7.3)	E332A (pH 7.3)
Muc5AC-3	***V_{max} (μM/(μmole*min))***	77867927.67 ± 2949326.67	418263.33 ± 70275.33	795415.67 ± 59855.33	4818831 ± 784217
	K_M (μM)	384.2 ± 55.0	197.3 ± 69.5	22.77 ± 6.38	73.41 ± 32.67
	k_{cat} (min^{-1})	1946.70 ± 73.73	10.46 ± 1.76	19.89 ± 1.50	120.48 ± 19.60
	k_{cat}/K_M ($(μmole*min)^{-1}$)	5.07 ± 1.34	0.053 ± 0.025	0.87 ± 0.23	1.64 ± 0.60

Table S4: Data collection and refinement statistics (Apo, Mn^{2+} soak, and pH 7.3 structures)

	TxgGalNAc-T3: Apo, pH 9.5	TxgGalNAc-T3: Mn^{2+} soak, pH 9.5	TxgGalNAc-T3: Muc5AC-3,13, Mn^{2+}, UDP-GalNAc-F, pH 7.3
	PDB ID **8UHV**	PDB ID **8UHZ**	PDB ID **8UI1**
**Data collection**			
Energy (λ)	1 Å	1 Å	1 Å
Space group	P 2 21 21	P 2 21 21	P 2 21 21
Molecules/asymmetric unit	1	1	1
Cell dimensions			
a, b, c (Å)	60.238, 67.281, 164.39	62.627, 65.012, 166.633	62.43, 65.713, 166.314
α, β, γ (°)	90, 90, 90	90, 90, 90	90, 90, 90
Resolution (Å) [1]	29.6 - 2.90 (3.01 - 2.90)	29.7 - 2.57 (2.61 - 2.57)	29.7 - 2.91 (3.01 - 2.91)
R_{pim} [1]	0.077 (0.482)	0.066 (0.458)	0.074 (0.618)
$I/\sigma I$ [1]	9.7 (1.67)	11.3 (1.6)	11.8 (1.08)
$CC^{1/2}$	0.991 (0.554)	0.987 (0.605)	0.986 (0.297)
Completeness (%) [1]	100 (100)	99.8 (97)	98 (96.5)
Redundancy [1]	7.4 (7)	7.2 (3.7)	6.7 (4.5)
No. unique			
reflections [1]	15357 (726)	22636 (1082)	15343 (727)
**Refinement**			
R_{work} / R_{free}	18.0/ 23.7	17.9/ 23.6	18.5/ 22.7
No. atoms			
Protein	8364	8277	8365
Mn^{2+}	0	3	2
UDP	0	0	36
Peptide / 2-acetamido-2-deoxy-β-D-galactopyranose	0	0	0
Water / Solvent	25/132	125/160	84/87
Average B-factors	53.42	42.32	60.23
Macromolecules	50.92	42.07	58.29
Solvent[2]	31.50	37.32	51.7
Ligands	69.22	65.72	76.71
R.m.s deviations			
Bond lengths (Å)	0.003	0.005	0.007
Bond angles (°)	0.562	0.65	0.73

[1] Data in the highest resolution shell is shown in the parenthesis.

[2] Water, Di(HydroxyEthyl)Ether and glycerol
