# Supplementary material for: A Toxoplasma gondii O-glycosyltransferase that modulates bradyzoite cyst wall rigidity is distinct from host homologues
Source: Nat Commun. 2024 May 6;15:3792. doi: 10.1038/s41467-024-48253-w (PMC11074326; doi:10.1038/s41467-024-48253-w)
Supplement: Supplementary file 3 — Description of Additional Supplementary Files [file 41467_2024_48253_MOESM3_ESM.docx]

Description of additional supplementary files

Supplementary dataset 1

Title: Quantum Chemistry Coordinate files

Description: These files contain the coordinates for the Fig.3d-f, where e and f coordinates were generated by Quantum Chemistry calculations at pH 7 (Fig. 3e) and at an artificially low pH (Fig. 3f).

Supplementary dataset 2

Title: Computer code

Description: Figure 5a Codes for Image Quantification

Supplementary dataset 3

Title: Plasmids and primers

Description: A folder containing plasmids, gRNA, and primers used for making variants of TxgGalNAc-T3 for in vitro and in vivo assays.
