# Supplementary material for: A Toxoplasma gondii O-glycosyltransferase that modulates bradyzoite cyst wall rigidity is distinct from host homologues
Source: Nat Commun. 2024 May 6;15:3792. doi: 10.1038/s41467-024-48253-w (PMC11074326; doi:10.1038/s41467-024-48253-w)
Supplement: Supplementary file 5 — Supplementary Data 2 [file 41467_2024_48253_MOESM5_ESM.docx]

## Jython Code for processing CST1 glycosylation quantification assay

from ij import IJ, WindowManager

from ij.io import DirectoryChooser

from ij.plugin.frame import RoiManager

import os

dc = DirectoryChooser("Choose your folder")

folder = dc.getDirectory()

if folder is None:

print("No folder selected.")

exit()

for file in os.listdir(folder):

if file.endswith("ch03.tif"):

# Reset ROI Manager for the current file

roiManager = RoiManager.getInstance()

if roiManager is not None:

roiManager.reset()

else:

roiManager = RoiManager()

filePathCh03 = os.path.join(folder, file)

filePathCh02 = os.path.join(folder, file.replace("ch03.tif", "ch02.tif"))

impCh03 = IJ.openImage(filePathCh03)

if impCh03 is None:

continue

IJ.run(impCh03, "Median...", "radius=2")

IJ.run(impCh03, "Subtract Background...", "rolling=50")

ip = impCh03.getProcessor()

ip.setAutoThreshold("MaxEntropy dark")

impCh03.setProcessor(ip)

IJ.run(impCh03, "Convert to Mask", "")

IJ.run(impCh03, "Analyze Particles...", "size=10-Infinity add")

impCh03.close()

impCh02 = IJ.openImage(filePathCh02)

if impCh02 is None:

continue

impCh02.show()

for i in range(roiManager.getCount()):

roi = roiManager.getRoi(i)

impCh02.setRoi(roi)

IJ.run(impCh02, "Measure", "")

impCh02.close() # Close the ch02 image

## R code for visualization and statistical analysis

#VISUALIZATION

library(tidyverse)

filename <- "ResultsBG.csv"

rawdf <- read.csv(filename, header = TRUE)

df <- rawdf %>%

mutate(genotype = case_when(

str_detect(Label, "wt") ~ "wt",

str_detect(Label, "333_") ~ "333",

str_detect(Label, "334_") ~ "334",

str_detect(Label, "336_") ~ "336",

str_detect(Label, "554_") ~ "554",

str_detect(Label, "617_") ~ "617",

str_detect(Label, "t2ko_") ~ "T2KO",

str_detect(Label, "t3ko") ~ "T3KO",

TRUE ~ NA_character_ ))

desired_order <- c("wt", "333", "334", "336", "554", "617", "T3KO", "T2KO")

df$genotype <- factor(df$genotype, levels = desired_order)

library(see)

max_value <- max(df$Mean, na.rm = TRUE)

ggplot(df, aes(x = genotype, y = Mean)) +

geom_violinhalf(trim = FALSE, fill = "grey", position = position_nudge(x = .2, y = 0)) +

geom_jitter(position = position_jitter(width = 0.1), color = "black", size = 1.5, alpha = 0.6) +

scale_y_log10(limits = c(1, max_value)) + # Transform the y-axis to a log scale. Adjust the minimum value

theme_bw() +

labs(x = "Genotype", y = "Mean Value", title = "Half Dot Plot and Half Violin Plot")

ggsave("violin_by_genotype.svg", width = 8, height = 6, dpi = 300, bg = "white")

# STATISTICAL ANALYSIS

library(stats)

library(multcompView)

anova_result <- aov(Mean ~ genotype, data = df)

summary(anova_result)

tukey_result <- TukeyHSD(anova_result)

print(tukey_result)

tukey_adj_p_values <- tukey_result[[1]][,4]

names(tukey_adj_p_values) <- rownames(tukey_result[[1]])

comp_letters <- multcompLetters(tukey_adj_p_values)

print(comp_letters)
