# Supplementary material for: A Toxoplasma gondii O-glycosyltransferase that modulates bradyzoite cyst wall rigidity is distinct from host homologues
Source: Nat Commun. 2024 May 6;15:3792. doi: 10.1038/s41467-024-48253-w (PMC11074326; doi:10.1038/s41467-024-48253-w)
Supplement: Supplementary file 7 — Reporting Summary [file 41467_2024_48253_MOESM7_ESM.pdf]

nature portfolio

Corresponding author(s): Nadine L. Samara

Last updated by author(s): Apr 16, 2024

Reporting Summary

Nature Portfolio wishes to improve the reproducibility of the work that we publish. This form provides structure for consistency and transparency in reporting. For further information on Nature Portfolio policies, see our Editorial Policies and the Editorial Policy Checklist.

Statistics

For all statistical analyses, confirm that the following items are present in the figure legend, table legend, main text, or Methods section.

n/a	Confirmed	
☐	☒	The exact sample size (*n*) for each experimental group/condition, given as a discrete number and unit of measurement
☒	☐	A statement on whether measurements were taken from distinct samples or whether the same sample was measured repeatedly
☐	☒	The statistical test(s) used AND whether they are one- or two-sided<br>*Only common tests should be described solely by name; describe more complex techniques in the Methods section.*
☒	☐	A description of all covariates tested
☒	☐	A description of any assumptions or corrections, such as tests of normality and adjustment for multiple comparisons
☐	☒	A full description of the statistical parameters including central tendency (e.g. means) or other basic estimates (e.g. regression coefficient) AND variation (e.g. standard deviation) or associated estimates of uncertainty (e.g. confidence intervals)
☐	☒	For null hypothesis testing, the test statistic (e.g. *F*, *t*, *r*) with confidence intervals, effect sizes, degrees of freedom and *P* value noted<br>*Give P values as exact values whenever suitable.*
☒	☐	For Bayesian analysis, information on the choice of priors and Markov chain Monte Carlo settings
☒	☐	For hierarchical and complex designs, identification of the appropriate level for tests and full reporting of outcomes
☒	☐	Estimates of effect sizes (e.g. Cohen's *d*, Pearson's *r*), indicating how they were calculated

Our web collection on statistics for biologists contains articles on many of the points above.

Software and code

Policy information about availability of computer code

Data collection	As described in Methods, all x-ay diffraction data were collected at the Argonne Photon Source. HKL2000 was used to process and scale the X-ray diffraction data (Otwinowski, Z. & Minor, W. Processing of X-ray Diffraction Data Collected in Oscillation Mode. Methods in Enzymology, C.W. Carter, Jr. & R. M. Sweet, Eds., Academic Press (New York). 276, 307-326 (1997)).
Data analysis	The initial structure was solved by molecular replacement using Phaser (CCP4) and an Alphafold2 model of T.gondii-GalNAc-T3 as an initial search model (Potterton, E., Briggs, P., Turkenburg, M. & Dodson, E. A graphical user interface to the CCP4 program suite. Acta Crystallogr D Biol Crystallogr 59, 1131-1137 (2003), Winn, M. D. et al. Overview of the CCP4 suite and current developments. Acta Crystallogr D Biol Crystallogr 67, 235-242 (2011), Jumper, J. et al. Highly accurate protein structure prediction with AlphaFold. Nature 596, 583-589 (2021)).<br><br>Initial models were rebuilt manually using Coot and refined in PHENIX (Emsley, P., Lohkamp, B., Scott, W. G. & Cowtan, K. D. Features and development of Coot. Acta Crystallogr D Biol Crystallogr 66, 486-501 (2010), Adams, P. D. et al. PHENIX: a comprehensive Python-based system for macromolecular structure solution. Acta Crystallogr D Biol Crystallogr 66, 213- 221 (2010)).<br><br>The final models were validated by using PROCHECK and MOLPROBITY (Laskowski, R. A., MacArthur, M. W., Moss, D. S. & Thornton, J. M. PROCHECK: a program to check the stereochemical quality of protein structures. Journal of Applied Crystallography 26, 283-291 (1993). https://doi.org:10.1107/s0021889892009944, Laskowski, R. A., Rulllman, J. A. C., MacArthur, M. W., Kaptein, R. & Thornton, J. M. AQUA and

PROCHECK-NMR: Programs for checking the quality of protein structures solved by NMR. Journal of Biomolecular NMR 8, 477-486 (1996)).

Structure figures were prepared with Pymol (The PyMOL Molecular Graphics System, Version 2.0 Schrodinger, LLC).

Biochemical data were analyzed using Prism Graphpad software.

Quantum chemical calculations were carried out with Gaussian 16 (Frisch, M. J. et al. Gaussian 16, Revision A.03. Gaussian, Inc., Wallingford CT (2016).

The computer code for analyzing data in Fig 5a is available as supplementary dataset 3: Jython code for processing CST1 glycosylation quantification assay.

For manuscripts utilizing custom algorithms or software that are central to the research but not yet described in published literature, software must be made available to editors and reviewers. We strongly encourage code deposition in a community repository (e.g. GitHub). See the Nature Portfolio guidelines for submitting code & software for further information.

Data

Policy information about availability of data

All manuscripts must include a data availability statement. This statement should provide the following information, where applicable:

- Accession codes, unique identifiers, or web links for publicly available datasets
- A description of any restrictions on data availability
- For clinical datasets or third party data, please ensure that the statement adheres to our policy

Structure coordinates and X-ray diffraction data have been deposited in the Protein Data Bank, www.wwpdb.org (PDB ID codes: 8UJG, 8UJH, 8UJF, 8UJE, 8UI6, 8UHV, 8UHZ, 8UI1). Source data are provided with this paper as a Source Data File and in Supplementary Dataset 2.

Research involving human participants, their data, or biological material

Policy information about studies with human participants or human data. See also policy information about sex, gender (identity/presentation), and sexual orientation and race, ethnicity and racism.

Reporting on sex and gender	N/A
Reporting on race, ethnicity, or other socially relevant groupings	N/A
Population characteristics	N/A
Recruitment	N/A
Ethics oversight	N/A

Note that full information on the approval of the study protocol must also be provided in the manuscript.

Field-specific reporting

Please select the one below that is the best fit for your research. If you are not sure, read the appropriate sections before making your selection.

[x] Life sciences [ ] Behavioural & social sciences [ ] Ecological, evolutionary & environmental sciences

For a reference copy of the document with all sections, see nature.com/documents/nr-reporting-summary-flat.pdf

Life sciences study design

All studies must disclose on these points even when the disclosure is negative.

Sample size	8 crystal structures were used in this manuscript. Each enzymatic assay was performed in duplicate or triplicate with measurements taken on distinct samples. 15 mutant T. gondii GalNAc-T3 enzymes were produced for enzymatic assays, as shown in Table S2. 6 novel T.gondii-GalNAc-T3 mutants were produced in vivo using the CRISPR/Cas9 system, and donor sequences and gRNA vectors are shown in supplementary files.
Data exclusions	Structural and enzymatic data are reported in Tables S1, S3, S4 and in Fig. 1, 2, 4, S2, S3, S7, and S9 without exclusion or modification.
Replication	The findings in this paper are reproducible. All biochemical data have duplicates or triplicates.
Randomization	N/A, not a human subject study or clinical trial.
Blinding	N/A, not a human subject study or clinical trial.

Reporting for specific materials, systems and methods

We require information from authors about some types of materials, experimental systems and methods used in many studies. Here, indicate whether each material, system or method listed is relevant to your study. If you are not sure if a list item applies to your research, read the appropriate section before selecting a response.

Materials & experimental systems

n/a	Involved in the study
☐	☒ Antibodies
☐	☒ Eukaryotic cell lines
☒	☐ Palaeontology and archaeology
☒	☐ Animals and other organisms
☒	☐ Clinical data
☒	☐ Dual use research of concern
☒	☐ Plants

Methods

n/a	Involved in the study
☒	☐ ChIP-seq
☒	☐ Flow cytometry
☒	☐ MRI-based neuroimaging

Antibodies

Antibodies used

Rabbit anti-GFP antibody (ThermoFisher #G10362), GalNAc glycoepitope-specific anti-CST1 antibody, HRP-conjugated rat anti-HA antibody (clone 3F10, Roche)

Validation

The references and websites below provide additional information on the antibodies used in this study:

https://www.thermofisher.com/antibody/product/GFP-Antibody-Recombinant-Monoclonal/G10362

Tomita, T. et al. The Toxoplasma gondii cyst wall protein CST1 is critical for cyst wall integrity and promotes bradyzoite persistence. PLoS Pathog 9, e1003823 (2013)

https://www.sigmaaldrich.com/US/en/product/roche/roahaha

Eukaryotic cell lines

Policy information about cell lines and Sex and Gender in Research

Cell line source(s)

human foreskin fibroblasts (HFF) cells (https://www.atcc.org/products/crl-1634)

Toxoplasma gondii: Prugniaud strain with deletion in KU80 and HXGPRT gene
Fox et al. 2011 (https://pubmed.ncbi.nlm.nih.gov/21531875/)

Authentication

The T. gondii line has been checked for a GFP signal under bradyzoite condition.

Mycoplasma contamination

HFF was routinely checked for the absence of Mycoplasma contamination by PCR. The cell line tested negative for contamination.

Commonly misidentified lines
(See ICLAC register)

N/A

Plants

Seed stocks

Report on the source of all seed stocks or other plant material used. If applicable, state the seed stock centre and catalogue number. If plant specimens were collected from the field, describe the collection location, date and sampling procedures.

Novel plant genotypes

Describe the methods by which all novel plant genotypes were produced. This includes those generated by transgenic approaches, gene editing, chemical/radiation-based mutagenesis and hybridization. For transgenic lines, describe the transformation method, the number of independent lines analyzed and the generation upon which experiments were performed. For gene-edited lines, describe the editor used, the endogenous sequence targeted for editing, the targeting guide RNA sequence (if applicable) and how the editor was applied.

Authentication

Describe any authentication procedures for each seed stock used or novel genotype generated. Describe any experiments used to assess the effect of a mutation and, where applicable, how potential secondary effects (e.g. second site T-DNA insertions, mosiacism, off-target gene editing) were examined.
